# Fifty years of *Nucleic Acids Research*

**DOI:** 10.1093/nar/gkad1156

**Published:** 2024-01-05

**Authors:** Julian E Sale, Barry L Stoddard

**Affiliations:** MRC Laboratory of Molecular Biology, Francis Crick Avenue, Cambridge CB2 0QH, UK; Division of Basic Sciences, Fred Hutchinson Cancer Center, 1100 Fairview Ave. N., Seattle WA 98109, USA

This month marks the 50th anniversary of *Nucleic Acids Research (‘NAR’)*. Led by a team of three founding editors (Richard T. Walker and A. Stanley Jones at the University of Birmingham in the UK and Dieter Söll at Yale University in the USA), the journal was formed for the publication of ‘original papers dealing with the physical, chemical, biochemical, biological or medical properties of nucleic acids, their constituents and analogs’ (1). The introduction to the inaugural issue went on to state that the journal's mission would be to promote ‘interdisciplinary means of communication that will enable scientists in one specialty to see more clearly the points of contact and importance between their work and that of others.’ That first issue featured fifteen research articles, spanning studies of DNA polymerase mechanism (2), tRNA modification and reactivity (3,4), DNA methylation (5) and ribosome assembly (6,7). Starting a trend that continues in the present-day journal, several additional papers described new methods, for the study of nucleic acid sequence composition, labeling or synthesis (8–10).

Now, a half-century later, *Nucleic Acids Research* stands as a leading journal in the biological sciences, publishing research studies describing the physical, chemical, biochemical, and cellular aspects of nucleic acids and of the proteins and effectors involved in their metabolism, modification, interactions, and biology. The journal also serves as the premier platform for the description and organization of online biological databases and webservers (11,12), and is a leader in the publication of research describing the development and use of nucleic acid therapeutics (13). Over the decades, *Nucleic Acids Research* has published and promoted many studies in areas that have fundamentally transformed molecular biology and biomedical research, including the biological role, development and application of restriction endonucleases (14) and CRISPR-based gene targeting systems (15), the characterization of complex DNA and RNA elements such as G-quadruplexes (16–18), the genesis and molecular behavior of noncoding RNA species such as microRNAs (19), and the mechanism and regulation of protein translation (20) – that latter content including a 2010 description of the effect of incorporation of pseudouridine in messenger RNA on immunogenicity and protein expression (21) that was cited by the Nobel prize committee as a seminal study leading to the development of mRNA vaccines (https://www.nobelprize.org/prizes/medicine/2023/press-release/).

Since its inception, *Nucleic Acids Research* has built its reputation as a leading journal in the molecular and cellular biological sciences not only by publishing the highest possible quality of original research, but also by embracing and promoting the advent and wide-spread influence of information technology throughout biological research, most importantly the creation of the world wide web and online platforms for data deposition, curation, organization, and dissemination. January 1996 saw the publication of the journal's first special issue dedicated solely to the development and role of online scientific databases, with 58 articles describing resources as diverse and as fundamentally important as GenBank (22), FlyBase (23), the RNA modification database (24) and the BLOCKS protein alignment database (25). Joined in 2003 by a separate annual issue devoted to Webservers ‘that perform useful computations on DNA, RNA and protein sequences and structures’ (26), the journal now publishes descriptions of over 250 database and webservers each year in two special online issues, and also maintains a curated online catalogue of databases to the benefit of the research community (http://www.oxfordjournals.org/nar/database/c/).

NAR also strives to be a leader in the free and open communication and accessibility of scientific research. In 2005, the journal (with the encouragement and participation of our publisher, Oxford University Press) was one of the first traditional print journals to adopt a fully open-access publication model that eliminated all barriers relating to paid subscriptions (27). The journal has also been an early and consistent advocate for deposition and immediate public availability of the data and results underlying our published content: for example, the journal announced the mandatory deposition and immediate release of PDB coordinates in January of 1992 (28) – well before most other journals and publishers instituted a similar requirement. More recently, with the advent of preprint servers and their role in the rapid, open dissemination of scientific data and results throughout the research community, the journal has adopted and promoted a policy encouraging the posting of studies to preprint servers prior to submission to our journal, as well as the citation of preprints within the journal's published content (29).

*Nucleic Acids Research* maintains a stated scope and criterion for its own published content that is primarily focused on mechanistic details of how interactions between nucleic acids and cellular factors drive biology. However, the journal recognizes that the studies published within its pages are intimately associated with equally important discovery-based, observational, and/or clinically focused research, much of which is focused on human disease and opportunities for therapeutic development. Therefore, the journal's leadership and publisher have fostered several partner journals that cater to informatic analyses and ‘big data’ (*NAR Genomics and Bioinformatics* (30)), neoplasia (*NAR Cancer* (31)) and molecular studies of human disease, including clinical medicine, pathology and drug discovery (*NAR Molecular Medicine;*https://academic.oup.com/narmolmed). The editorial leadership of each of these journals work closely with NAR and with one another to ensure that relevant submissions to our own journal are rapidly assessed for suitability in any of these related journals.

As we move forward into the second half of our first century in press, the leadership of *Nucleic Acid Research* is obviously proud of the journal's accomplishments and reputation, as reflected in its consistent ranking as one of the top scientific research journals in the world (https://scholar.google.com/citations?view_op=top_venues). However, what continues to be our most important goal, and the source of greatest pride, is that *Nucleic Acids Research* remains a research journal that is run by active research scientists, entirely for the benefit of research scientists. The leadership of the journal, starting with the Senior Editors and the Executive Editorial team, along with an outstanding editorial board and extended peer review community, have dedicated themselves to pursuing rapid, transparent, rigorous and fair management of the publication process, from initial queries from potential authors, to subsequent peer review and revisions, and finally the timely handling of any responses to that content that result in revisiting, updating, modifying or correcting our content.

As part of our fiftieth anniversary celebration, we are establishing an Early Career Researcher Advisory Board. Individuals on the ECR board will not only provide input on journal strategy but also have the opportunity to gain experience in aspects of running the journal, including editorial and peer review processes. We hope this initiative will help nurture the next cohort of practicing scientists who contribute to the endeavor of top tier scientific publishing.

At this time of celebration of a half-century of outstanding science described in the pages of *Nucleic Acids Research*, the entire editorial and publishing team look forward to many more years of working with research investigators and authors around the world. We conclude with a heart-felt thanks to all the individuals (editors, authors, reviewers, administrators, and publishers) who have, since the earliest days of modern molecular biology and our journal's start, made *Nucleic Acids Research* well-worth every bit of the considerable amount of energy, time and dedication required to run one of molecular and cellular biology's leading research journals.
